# Different mechanisms are implicated in *ERBB2* gene overexpression in breast and in other cancers

**DOI:** 10.1038/sj.bjc.6601200

**Published:** 2003-08-26

**Authors:** D Vernimmen, M Gueders, S Pisvin, P Delvenne, R Winkler

**Affiliations:** 1 Molecular Oncology Laboratory, Experimental Cancer Research Center, Liège University, B-4000 Liège, Belgium; 2 Pathology Department, Experimental Cancer Research Center, Liège University, B-4000 Liège, Belgium

**Keywords:** immunocytochemistry, *ERBB2* promoter, AP-2 transcription factor, human

## Abstract

The *ERBB2* gene is overexpressed in 30% of breast cancers and this has been correlated with poor prognosis. *ERBB2* is upregulated in other cancers such as prostate, pancreas, colon and ovary. In breast cancer cells, the mechanisms leading to *ERBB2* gene overexpression are increased transcription and gene amplification. In these cancers, AP-2 transcription factors are involved in *ERBB2* overexpression, and AP-2 levels are correlated with p185^c-*erb*B-2^ levels. In this work, we wanted to know if the same molecular mechanisms are responsible for the *ERBB2* upregulation in non-breast cancers. We compared *ERBB2* gene copy number, p185^c-*erbB*-2^ and mRNA levels with AP-2 levels in several ovary, prostate, colon and pancreas cancer cells. A moderate expression of *erb*B-2 mRNA and protein were observed in some cells without gene amplification. In contrast to breast cancer cells, AP-2 factors were absent or low in some non-breast cells which did express *ERBB2*. It is thus likely that AP-2 is not a major player in the increased levels of *erb*B-2 transcripts in non-breast cancer cells. The transcriptional activity of the *ERBB2* promoter in colon and ovary cancer cells was estimated using reporter vectors. The results showed that the promoter regions involved in *ERBB2* gene overexpression in breast cancer cells are different from those that lead to the gene upregulation in colon and ovary cancers. In conclusion, our results indicate that different transcriptional and post-transcriptional mechanisms are responsible for the increased levels of *erb*B-2 transcript and protein in breast and non-breast cancer cells.

The *ERBB2* gene is located on chromosome 17q21 and encodes a 185 kDa transmembrane receptor (p185^c-*erb*B-2^) belonging to the EGFR family (Yarden and Sliwkowski, 2001). *ERBB2* is overexpressed in about 30% of breast and ovary cancers and this has been correlated with poor prognosis for the patient (Ross and Fletcher, 1999). Recently, the use of a p185^c-*erb*B-2^ targeted antibody, trastuzumab (Herceptin), has been used successfully to inhibit the growth of breast cancer cells.

Most of the studies aimed at understanding the molecular basis of *ERBB2* overexpression have been performed in breast cancers. In these cancers, the molecular mechanisms involved are transcriptional upregulation and gene amplification (Hollywood and Hurst, 1993; Pasleau *et al*, 1993). AP-2 and Ets family transcription factors have been shown to contribute to *ERBB2* overexpression in breast cancer cells. The AP-2 transcription factors upregulate the *ERBB2* expression by binding to two sites located −213 bp (Bosher *et al*, 1996) and −500 bp upstream the Cap site (Vernimmen *et al*, 2003). The Ets transcription factor stimulates *ERBB2* expression by preventing the formation of a triplex structure on the core promoter (Scott *et al*, 2000).

The *ERBB2* gene is also overexpressed in other cancers such as prostate, colon and pancreas cancers, and this alteration has also been considered as a negative prognosis marker by some authors (Klapper *et al*, 2000).

In this report, we address, for the first time, the molecular mechanisms leading to *ERBB2* gene overexpression in non-breast cancer cells. We wanted to know if the mechanisms, which have been shown to operate in breast cancers, are also at work in non-breast cancers. For this purpose, we used cell lines derived from ovary, prostate, colon, liver and pancreas cancers. First, we compared the *ERBB2* gene copy number, mRNA and protein levels. The p185^c*-erb*B-2^ levels were estimated by immunocytochemistry (ICC) and Western blotting. The *ERBB2* gene copy numbers and mRNA levels were measured by real-time PCR. In parallel, we assessed the AP-2 levels and DNA binding activities in all these cell lines. The results showed a moderate *ERBB2* overexpression in a significant proportion of non-breast cancer cells. Contrary to the results obtained in breast cancer cell lines, there was no correlation between the levels of the *erb*B-2 mRNA and the AP-2 transcription factor. We then analysed the *ERBB2* promoter activity in the different cell lines by transfecting reporter vectors containing progressive deletions of a 6 kb promoter (Grooteclaes *et al*, 1994). The transcriptional activity increased with increasing sizes of the *ERBB2* promoter. Nevertheless, the regulatory fragments we identified in breast cancer cells (Grooteclaes *et al*, 1994) function differently in non-breast cancer cells. In conclusion, the accumulation of *erb*B-2 mRNA and protein in breast and non-breast cancer cells are the consequences of different transcriptional and/or post-transcriptional events.

## MATERIAL AND METHODS

### Cell lines

The mammary (BT-474, ZR-75.1 and MDA-MB-231), hepatic (HepG2), prostatic (LNCaP, DU 145 and PC-3), colon (WiDr, HTm29, HCT 116, COLO 205 and COLO 320), ovary (OVCAR-3 and SK-OV-3) and pancreatic (PANC-1, Miapaca-2, HS766 T, CF-PAC-1, SU.86.86, BxPC-3 and Capan-2) human epithelial cells were purchased from American Type Culture Collection (Manassas, VA, USA) and cultured in the recommended media supplemented with 10% fetal bovine serum, 2 mM glutamine and 100 *μ*g ml^−1^ penicillin/streptomycin. (Biowhittaker, Walkersville, MD, USA).

### Immunocytochemistry

Cells (50 × 10^6^) were harvested by trypsinisation and centrifugation. After centrifugation, the cell pellets were fixed in 2% paraformaldehyde (UCB, Louvain, Belgium), then embedded in paraffin. Sections (5 *μ*m thick) were deparaffinized and rehydrated using xylene and graded alcohols. The sections were heated at 100°C for 40 min in a citrate buffer, then incubated for 20 min at room temperature. Endogenous peroxidase activity was blocked with 5% H_2_O_2_ for 5 min. After two washes, for 5 min each, with 1% tween-phosphate-buffered saline (PBS) solution, the sections were incubated with an antibody diluent solution (Dako Diagnostics, Glostrup, Denmark) containing a c-*erb*B-2 monoclonal antibody (1 : 300) raised against the internal domain of the p185^c-*erb*B-2^ protein (NCL-CB11, Novocastra, Newcastle, UK). Anti-mouse HRP-labelled polymer (Dako) was applied for 30 min at room temperature and the slides were washed for 2 × 5 min with 1% tween-PBS solution. The sections were then incubated for 40 min with DAB^+^ substrate (Dako), washed three to four times in water and counterstained with haematoxylin. Cytoplasmic and membrane immunostaining was evaluated using a 0 to 3+ scale (0, negative or equivocal positivity; 1+, weak positivity; 2+ moderate positivity; 3+ strong positivity).

### Real-time PCR and real-time RT–PCR

Genomic DNA was extracted by the phenol–chloroform procedure (Maniatis *et al*, 1982). Total cellular RNA was extracted with the Tripure Isolation Reagent (Roche Diagnostic, Basel, Switzerland). DNA and RNA quantification were performed with the LightCycler–HER2/neu DNA and RNA Quantification Kits (Roche).

### Electromobility shift assays (EMSA)

Nuclear extracts, HTF/AP-2 *cis* sequence and EMSA were described elsewhere (Schreiber *et al*, 1989; Vernimmen *et al*, 2003). Briefly, 2–4 *μ*g of crude nuclear proteins were incubated with 300 000 c.p.m. of [*α*-^32^P]dCTP end-labelled oligonucleotide. The retarded complexes were analysed on a nondenaturing 5% polyacrylamide gel and analysed using a PhosphorImager (Molecular Dynamics Amersham Biosciences, Roosendal, The Netherlands).

### Western blotting

For p185^c-*erb*B-2^ detection, cells were scraped off the culture dishes, harvested in PBS, pelleted by centrifugation, resuspended in a 1% SDS solution and boiled for 10 min. Whole cell extracts (20 *μ*g) were loaded per well, separated on a 12% SDS–polyacrylamide gel and transferred to a PVDF membrane (Millipore, Brussels, Belgium). A c-*erb*B-2 antibody (06-562 Euromedex, Mundolsheim, France) was used at a 1 : 2000 dilution. For AP-2 detection, 10–25 *μ*g of nuclear extracts were loaded per well. An AP-2*α* antibody (sc-184 Santa Cruz Biotechnology, Santa Cruz, CA, USA) was used at a 1 : 700 dilution. Secondary antibodies (Dako Diagnostics, Glostrup, Denmark) were detected with the ECL system (Amersham BioSciences). The *β*-actin monoclonal antibody was from Sigma (monoclonal (amoeba) mouse ascites fluid clone KJ43A Sigma-Aldrich Bernem, Belgium).

### Plasmids and transient transfection assays

The transfection efficiencies of all the cell lines were tested by transfection of the pEGFP-IRESpuro control vector (Clontech, Palo Alto, CA, USA). Cells were transfected using the FuGENE 6 reagent (Roche). Cells (4 × 10^5^) were plated on 35 mm tissue culture dishes with a FuGENE/DNA ratio of 3 : 1. The cells were incubated for 48 h in complete medium. Cells transfected with the green fluorescent protein (GFP) expression plasmid were visualised by fluorescent microscopy. The luciferase (LUC) reporter vectors containing different *ERBB2* promoter fragments have been previously described (Grooteclaes *et al*, 1994). The LUC enzymatic activities were measured using the Luciferase Reporter Gene Assay kit (Roche).

## RESULTS

### ErbB-2 gene copy number, mRNA and protein levels in cancer cell lines

**Figure 1:**
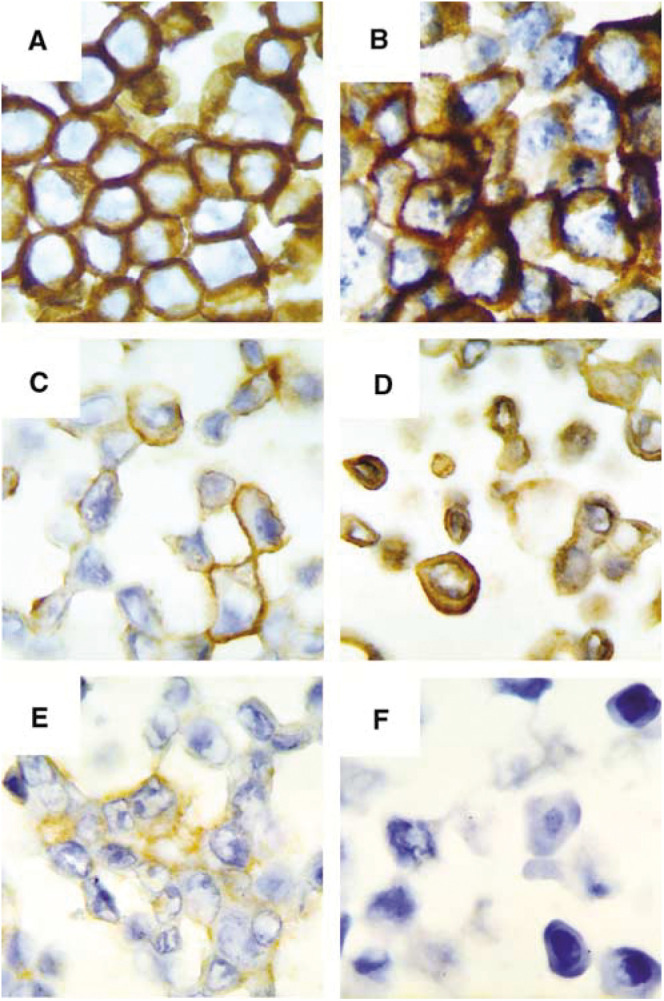
Representative examples of p185^c-*erb*B-2^ immunostaining in cancer cell lines. (**A**) BT-474 (mammary cell line with *ERBB2* amplification and 3+ membrane overexpression). (**B**) SK-OV-3 (ovary cell line with *ERBB2* amplification and 3+ membrane overexpression). (**C**) ZR-75.1 (mammary cell line without *ERBB2* amplification and 2+ membrane overexpression). (**D**) HTm29 (colon cell line without *ERBB2* amplification and 2+ cytoplasmic/1+ membrane overexpression). (**E**) HepG2 (liver cell line without *ERBB2* amplification and 2+ cytoplasmic overexpression). (**F**) SU.86.86 (pancreatic cell line without *ERBB2* amplification and no cytoplasmic/membrane expression)(magnification X400).

We measured the p185^c-*erb*B-2^ protein levels by ICC and Western blotting. For ICC, we used the well characterised breast cancer cell lines BT-474 and ZR-75-1 as standards to determine the *erb*B2 expression in the non-breast cells (Figure 1A, C

). After ICC, p185^c-*erb*B-2^ appeared as a brown membrane staining in positive breast cancer cells. Likewise, p185^c-*erb*B-2^ exhibited an intense membranous staining in SK-OV-3 ovary carcinoma cells (Figure 1B). P185^c-*erb*B-2^ was detected in one out of two ovary, four out of five colorectal, three out of three prostatic and only two out of seven pancreatic cancer cells, but the staining was heterogeneous and mainly cytoplasmic (Figure 1D and Table 1

**Table 1:** *ERBB2 gene amplification, mRNA and protein levels in cancer cell lines*.

**Origin**		**Breast**		**Ovary**		**Prostate**			**Colon**					**Liver**	**Pancreas**						
**Cell line**		**MDA-MB-231**	**BT–474**	**OVCAR-3**	**SK-OV-3**	**DU 145**	**PC-3**	**LNCaP**	**HTm29**	**WiDr**	**HCT116**	**COLO 205**	**COLO 320**	**HepG2**	**SU.86.86**	**B × PC-3**	**HS766T**	**PANC-1**	**CF-PAC-1**	**Miapaca-2**	**Capan-2**
ICC	M	0	3+	0	3+	0	0	0	1+	0	0	0	0	0	0	0	0	0	0	0	0
	C	0	0	0	0	1+	1+	2+	2+	1+	0	1+	2+	0	0	0	0	1+	0	1+	
Wb	Protein	1	15*	2	13*	1.5	1	7.5	2	1	1	3	1	11	0.10	0.05	0.15	0.25	2.10	1.60	2.30
RT–PCR	RNA	1	70	1	60	0.2	0.3	2.2	0.5	1	2	2	6.5	6.5	0.04	0.06	0.04	0.05	1	1.1	1.7
PCR	DNA	1	8	1	4	1	1	1	1	1	1	1	1	1	1	1	1	1	1	1	1

The membrane (M) and cytoplasmic (C) staining (IHC) of p185^erbB−2^ is expressed as values comprised between 0 (no staining) and 3+ (intense staining). Western blotting (Wb) was performed using 20 *μ*g of whole cell extracts. Expression patterns were obtained by comparing the p185^erbB−2^ and *β*-actin levels in each cell line. *ERBB2* mRNA levels were measured by real–time RT–PCR and compared to G6PD levels as the internal control for each cell line. The values for mRNA and protein levels measured in all the cell lines were referred to the values in MDA-MB-231 cells considered as equal to 1. Gene copy number was estimated by real–time PCR. The values marked by an asterisk are underestimated due to the saturation of the film.

). P185^c-*erb*B-2^ was also detected in the cytoplasm of HepG2 hepatocarcinoma cells (Figure 1E). Figure 1F presents a pancreatic cell line negative for p185^c-*erb*B-2^.

The p185^c-*erb*B-2^ levels were also estimated by Western blotting of whole-cell extracts (Table 1 and Figure 4). As cell density has been reported to modulate p185^c-*erb*B-2^ levels in breast cancer cells (Kornilova *et al*, 1992), we compared the oncoprotein levels in low (50% confluence)- and high (100% confluence)-density cultures. The full-length, 185 kDa protein, was detected in most analysed cells. A slight difference was observed between low- and high-density cultures of breast, ovary and pancreatic cell lines (Figure 4). The highest p185^c-*erb*B-2^ levels were observed in BT-474 breast and SK-OV-3 ovary cancer cells (Table 1). In order to compare the protein content between the different cancer types, we attributed the value of one to the p185^c-*erb*B-2^ measured in MDA-MB-231 mammary cancer cells (Table 1). BT-474 and SK-OV-3 cells contained the highest protein levels associated with gene amplification and mRNA overexpression. Among the cells without gene amplification, HepG2 hepatocarcinoma and LNCaP prostate cancer cells were most enriched in p185^c-*erb*B-2^. All the colon cancer cell lines contained almost similar protein levels not significantly different from that of MDA-MB-231 cells. The pancreatic cell lines SU.86.86, BxPC-3, HS766 T and PANC-1 contained very low levels of p185^c-*erb*B-2^, detectable only after long exposure time. Only CF-PAC-1, Miapaca-2 and Capan-2 cells attained or slightly exceeded the MDA-MB-231 p185^c-*erb*B-2^ levels. Notice the wide variation in p185^c-*erb*B-2^ between the pancreatic cancer cells. In general, Western blotting and ICC results were in reasonably good agreement (Table 1).

The *erb*B-2 mRNA levels were measured by real-time RT–PCR. The results are summarised in Table 1 and compared to the Western blotting data. In most cells, real-time RT–PCR and Western blotting data were in good agreement, except for the COLO 320 cancer cells. Indeed, in these cells, the increase in transcript levels was not accompanied by an increase in protein levels (Table 1). Like the protein levels, *erb*B-2 mRNA levels were quite high in HepG2 cells.

The *ERBB2* gene copy numbers were estimated by real-time PCR in all cancer cells (Table 1). The gene was not amplified in any of the prostate, colon and pancreatic cancer cells. SK-OV-3 presents a four-fold amplification of the *ERBB2* gene.

### AP-2 transcription factors are not involved in ERBB2 overexpression in non-breast cancer cells

**Figure 2:**
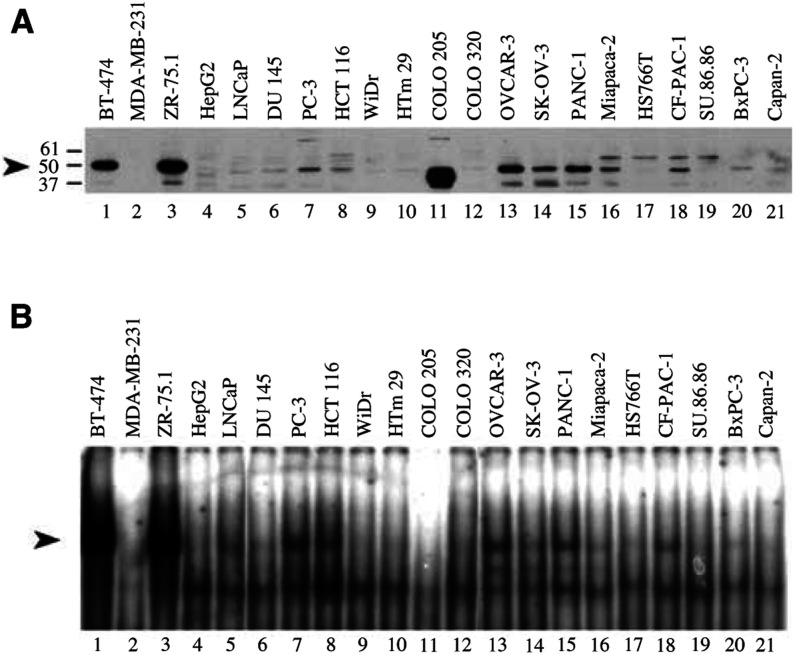
Distribution of AP-2 transcription factor in non-breast cancer cell lines. (**A**) Western blot with10 *μ*g (control, lanes 1 and 3) or 20 *μ*g (lanes 2 and 4–21) of crude nuclear extracts from breast and non-breast cells. The arrowhead marks the 50 kDa molecular weight of AP-2 proteins. (**B**) EMSA assays with 2 *μ*g (lanes 1 and 3) or 4 *μ*g (lanes 2 and 4–21) of crude nuclear extracts. The arrowhead marks specific AP-2/DNA complex.

AP-2 levels are high in most breast cancer cells overexpressing *ERBB2* (Bosher *et al*, 1996). To find out if these transcription factors are also involved in the stimulation of *ERBB2* expression in non-breast cancer cells, we compared AP-2 and *erb*B-2 levels in the cell lines used in this study. AP-2*α* levels were estimated by Western blotting (Figure 2A

) and AP-2 DNA binding activity was analysed by gel retardation experiments (EMSA) (Figure 2B). BT-474 and ZR-75.1 breast cancer cells were used as AP-2-positive controls and HepG2 hepatocarcinoma cells and MDA-MB-231 breast cancer cells as AP-2-negative controls (Bosher *et al*, 1996). AP-2 levels were very low in colon cancer cells (Figure 2A, lanes 8–12). Interestingly, in COLO 205 cells, the AP-2 antibody revealed three intense bands of lower molecular weight (Figure 2A, lane 11). No DNA binding activity was detected in these cells (Figure 2B, lane 11), suggesting that this might be a false positive signal. Low levels of the 50 kDa AP-2 factor were detected in prostate cancer cells (Figure 2A, lanes 5–7). In the two ovary cancer cell lines, AP-2*α* was easily detected (Figure 2A, lanes 13, 14). However, the signal was much less intense than in breast cancer cells overexpressing AP-2 (see for instance Figure 2A, lanes 1 and 3). Finally, in the pancreatic cells, the AP-2*α* levels were low to moderate. In these cells, a higher molecular weight band was detected together with the 50 kDa protein (Figure 2A, lanes 15–21). The Western blotting and the EMSA results showed comparable patterns in all the cell lines, indicating that when AP-2 was present, it binds efficiently to DNA.

In conclusion, there was no correlation between AP-2 and *erb*B-2 mRNA or protein levels in the non-breast cancer cell lines. The most striking discrepancy was observed with HepG2 cells, widely used as AP-2 negative controls, but these cells do express the *erb*B-2 mRNA and protein. Our results thus suggest that the AP-2 is not involved in *ERBB2* overexpression in the non-breast cancer cell lines we tested.

### ERBB2 promoter activity in non-breast cancer cells

**Figure 3:**
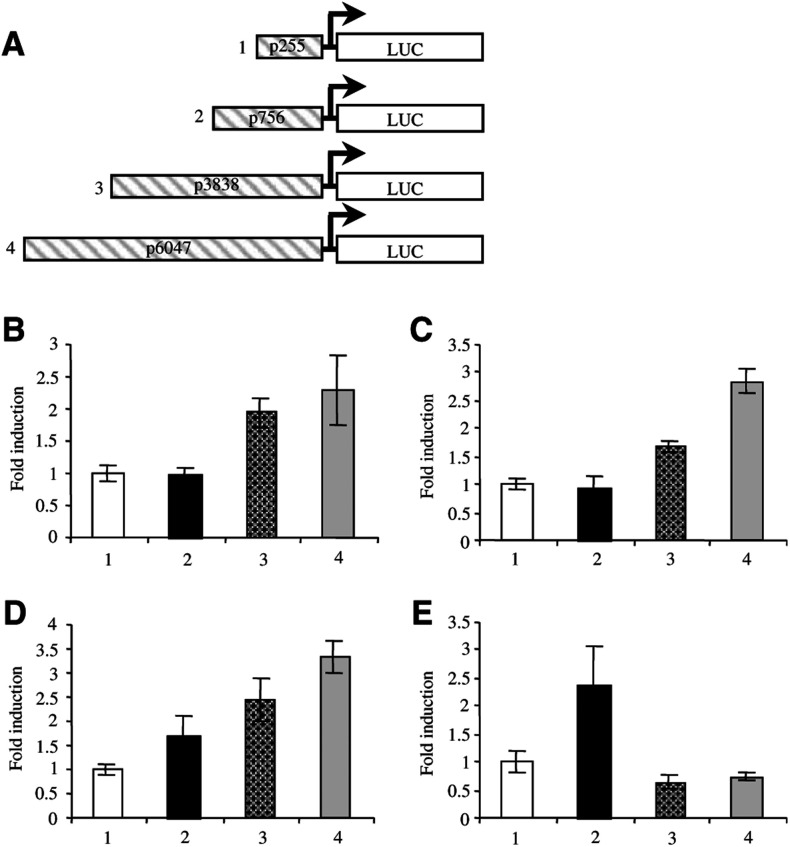
Luc assays using different *ERBB2* promoter constructs. (**A**) Illustration of the different reporter vectors used. The promoter fragment sizes are indicated in the hatched boxes. The luciferase activity was measured in colon HCT 116 (**B**), COLO 320 (**C**) and ovary OVCAR-3 (**D**) and SK-OV-3 (**E**) cancer cells. The results are expressed as fold induction compared to the level obtained with the p255-LUC vector. The data are shown as the mean ±s.d. of triplicate experiments.

In order to better understand the mechanisms leading to *ERBB2* overexpression in the non-breast cancer cells, we transfected reporter vectors containing progressive deletions of a 6 kb promoter fragment (Figure 3A

). We have shown previously that these promoter fragments are active in breast cancer cells (Grooteclaes *et al*, 1994). The transfection efficiencies of all the cell lines were estimated by transfection of an EGFP-expression vector (data not shown). Only the cells having a transfection efficiency of at least 5%, namely colon and ovary cancer cells, were used for further studies. The luciferase activity was compared in cell lines synthesising low *vs* high levels of the *erb*B-2 transcript (HCT 116 *vs* COLO 320; OVCAR-3 *vs* SK-OV-3). In both colorectal cell lines (HCT116 and COLO 320), the 6 kb promoter fragment induced a 2.5-fold increase of the LUC activity compared to the vector containing 200 bp of the promoter (Figure 3B, lane 4 and 3C, lane 4). The low-*ERBB2*-expressing ovary cancer cells, OVCAR-3, showed an increase of the LUC activity with the promoter size (Figure 3D). This increase was similar to that observed in the colorectal cells (compare Figure 3B–D). Only the p756-LUC construct increased the LUC activity in *ERBB2*-overexpressing SK-OV-3 cells (Figure 3E, lane 2). The longer promoter fragments downregulated the transcriptional activity to the basal value (Figure 3E, lanes 3 and 4). It is interesting to note that in this cell line the *ERBB2* gene is amplified four-fold and overexpressed more than 60 times (King *et al*, 1992 and personal results, Table 1).

## DISCUSSION

Around 30% of breast cancers overexpress the *ERBB2* gene and this is correlated with a poor prognosis. Besides gene amplification, several investigators have described the involvement of ETS family (Scott *et al*, 2000) and AP-2 family (Bates and Hurst, 1997) transcription factors in the gene overexpression. *ERBB2* overexpression has also been reported in cancers of colon (Nakae *et al*, 1993; Kapitanovic *et al*, 1994; Maurer *et al*, 1998), prostate (Ross *et al*, 1993; Morote *et al*, 1999), ovary (Fajac *et al*, 1995) and pancreatic (Yamanaka *et al*, 1993) origin. However, the mechanisms leading to increased expression of the gene have not been investigated in these tumours. Gene amplification is rare in these tumours and cannot account for the observed increase in the mRNA or protein levels. To our knowledge, this is the first attempt to understand the molecular mechanisms leading to *ERBB2* overexpression in non-breast cancers.

First, we assessed p185^c-*erb*B-2^ levels by ICC and by Western blotting. One of the main observations of this study is that breast and ovary cancer cell lines were characterised by a membrane homogeneous staining, whereas in the non-breast cancer cells, the staining was cytoplasmic and heterogeneous (Figure 1, Table 1). Several truncated intracellular p185^c-*erb*B-2^ fragments have been described (Scott *et al*, 1993; Esparis-Ongando *et al*, 1999; Christianson *et al*, 1998; Molina *et al*, 2002). These fragments, which signal actively, might be responsible for the cytoplasmic staining observed in most non-breast cancer cells. However, only the full-length protein was detected by Western blotting in these cells, which does not support the hypothesis that ICC detects a truncated cytoplasmic protein. The cytoplasmic staining of p185^c-*erb*B-2^ has already been reported in prostate cancers (Rosset *et al*, 1993). De Potter *et al* (1989) have described the presence of a protein on the mitochondrial membrane, appearing as granular staining in the cytoplasm. The significance of the cytoplasmic staining in breast cancer cells is controversial, since some authors did not observe a correlation between the cytoplasmic protein and the mRNA levels (DiGiovanna, 1999; Ross and Fletcher, 1999). However, in the non-breast cancer cells we have analysed, there was a good correlation between these parameters. The mechanism by which p185^c-*erb*B-2^ is mainly cytoplasmic is unknown. The protein might not be properly targeted to the membrane or, alternatively, might be internalised (DiGiovanna, 1999).

We have characterised the *ERBB2* gene copy number, mRNA and protein levels in the tumour cell lines investigated in this study. Gene amplification was detected only in BT-474, MDA-MB-453 and SK-OV-3 cell lines, which is in good agreement with already published data. LNCaP prostate carcinoma cells, three pancreatic cells (Miapaca-2, Capan-2 and CF-PAC-1) and three colon cancer cells (HCT116, COLO 205 and COLO 320) showed a significant increase in *erb*B-2 mRNA levels without gene amplification. Compared with breast cancer cells, the increase in the transcript levels in these cells was low to moderate. HepG2 hepatocarcinoma cells expressed quite high levels of *erb*B-2 mRNA and protein. Indeed, the mRNA level was about the same as in COLO 320 cells but the protein level was much higher than in colon cancer cells.

The increased levels of p185^c-*erb*B-2^ have been shown to affect the biology of the tumour. For instance, increased p185^c-*erb*B-2^ levels in prostate cancers were associated with the passage from the androgen-dependent to the hormone-independent status (Signoretti *et al*, 2000). *Erb*B2 might stimulate the proliferation of colon cancer cells by upregulating COX2 (Mann *et al*, 2001).

**Figure 4:**
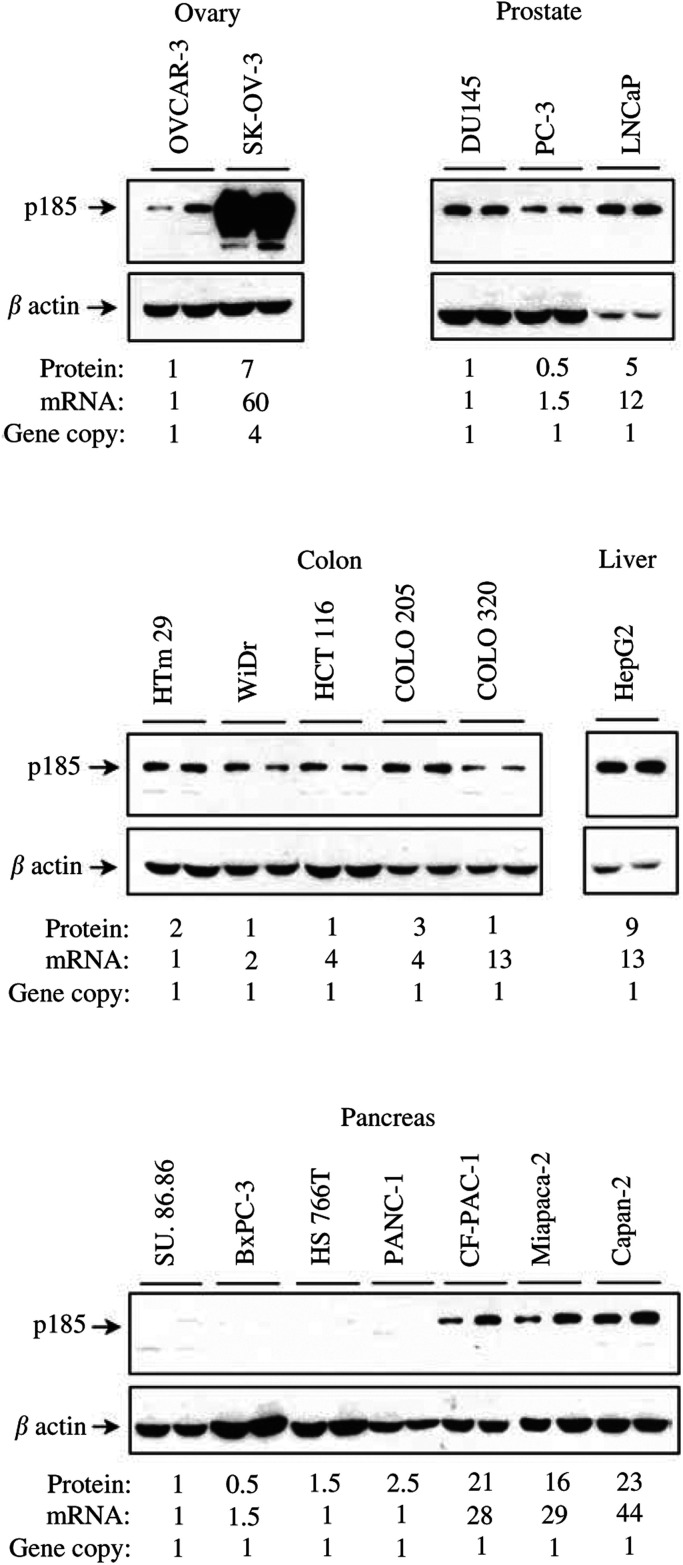
Expression of p185^*c-erb*B-2^ and *erb*B-2 messenger RNA in cancer cell lines. The first and the second lanes for each cell line correspond to pre-confluent and confluent cultures respectively. In order to compare the protein content in each cancer type, the value of one was given to the cell line expressing the lowest level of p185^*c-erb*B-2^ protein. The indicated values for protein and mRNA correspond to those measured for confluent cells.

In the ovary, prostate and pancreas cells, a good correlation was observed between the relative protein and mRNA levels (Figure 4

). On the contrary, in colon cancers, we observed an increase in mRNA levels while the protein levels were unchanged. We suggest two explanations for this discrepancy. First, the messenger RNA translation could be less efficient in colon cancer cells. Indeed, Child *et al* (1999) have shown that the *erb*B-2 transcript is translated with different efficiencies in different cell lines. Second, the protein half-life might be shorter in these cells. Future studies are needed to address these questions.

As a first approach to the understanding of *ERBB2* gene expression regulation in non-breast cancer cell lines, we compared *ERBB2* expression levels with AP-2*α* protein levels and with AP-2 DNA binding activity in these cells. As EMSA detects the binding of AP-*α*, *β* and *γ* transcription factors, this technique gives a more accurate picture of AP-2 transcriptional activity than the Western blotting. We analysed the possible involvement of AP-2 in the increased *ERBB2* expression, because of the well-characterised role of this transcription factor in breast cancer cells. Indeed, the 500 bp proximal *ERBB2* promoter contains two AP-2 binding sites, which contribute to the gene overexpression. The first site is located 215 bp upstream from the transcription initiation site (Bosher *et al*, 1995), whereas the second lies 501 bp upstream from the transcription start site (Grooteclaes *et al*, 1999; Vernimmen *et al*, 2003).

Nuclear AP-2*α* levels and AP-2 DNA binding activities were considerably lower in prostate, colon, ovary and pancreatic cell lines than in breast cancer cells. Since AP-2 could be cytoplasmic rather than nuclear (see below), we also analysed whole-cell extracts by Western blotting. We did not observe any difference between nuclear and total AP-2 levels, indicating that the low levels of the nuclear factor is not the consequence of its cytoplasmic retention (data not shown). Besides the full-length 50 kDa protein, we detected lower and higher molecular weight proteins as well. The lower molecular weight bands might result from the proteolysis of the transcription factor. Higher molecular weight immunoreactive forms were observed mainly in the pancreatic cells (Figure 2A) and might represent the recently described, 60 kDa sumolated form of the protein. The transcriptional activity of these sumolated factors is reduced (Eloranta and Hurst, 2002).

AP-2*α* levels were not correlated with *ERBB2* expression in non-breast cancer cell lines. This indicates that AP-2 is not involved in *ERBB2* gene overexpression. In other cancers, the role of AP-2 has been shown to vary according to the tumour type. Some authors described the loss of AP-2*α* expression early in the development of prostate adenocarcinoma (Ruiz *et al*, 2001). Moreover, AP-2B, a dominant-negative variant of AP-2*α*, was detected by RT–PCR in LNCaP cells (Buettner *et al*, 1993). Others did detect AP-2*α* by immunohistochemistry in some prostate cancer specimens (Lipponen *et al*, 2000). In primary colorectal (Ropponen *et al*, 2001) and ovarian cancers (Anttila *et al*, 2000), AP-2*α* was detected in the nucleus or the cytoplasm of tumour cells. However, the presence of the transcription factor in the cytoplasm precludes its function in transcription. Interestingly, high AP-2*α* mRNA levels were detected in some tumours which were negative for the protein (Karjalainen *et al*, 2000).

To analyse the molecular mechanisms leading to *ERBB2* overexpression in non-breast cancer cell lines, we transfected four *LUCIFERASE* reporter vectors containing 200 bp–6 kb fragments of the *ERBB2* promoter in colon and ovary cell lines. The relative transcriptional activity of each reporter was compared to the luciferase activity induced by the p255-LUC vector, considered as equal to one (Grooteclaes *et al*, 1994). In the colorectal cancer cell lines, the 700 bp fragment induced the same luciferase activity as the 200 bp promoter. The absence of AP-2 in these cells could explain the result. Indeed, we have shown that AP-2 is responsible for the transcriptional activating properties of this fragment in breast cancer cells (Grooteclaes *et al*, 1999; Vernimmen *et al*, 2003). The 6 kb promoter fragment induced a comparable increase in transcriptional activity in both colorectal cell lines. The three-fold difference in *erb*B-2 mRNA levels between HCT116 and COLO 320 cells is probably too small to be reflected by a significant difference in the promoter activity (Table 1). In SK-OV-3 ovary cancer cell line, overexpressing about 60 times the *erb*B-2 transcript, only the p756-LUC vector induced a significant increase in the transcription level (Figure 3E, compare lanes 1 and 2). The longer promoter fragments repressed the transcription. On the contrary, in OVCAR-3 cells, which do not overexpress *ERBB2*, we observed a progressive increase in transcriptional activity with longer promoter fragments. This result could be explained by the presence of a 8 kb transcript with an extended half-life in SK-OV-3 cells (Doherty *et al*, 1999a). Abnormal amounts of the *erb*B-2 mRNA could accumulate not only because of transcriptional upregulation, but also because of its stabilisation. We (Pasleau *et al*, 1993) and others (Hollywood and Hurst, 1993; Miller *et al*, 1994) have shown that mRNA stabilisation is not responsible for *ERBB2* overexpression in breast cancer cells. However, this is not necessarily true for other cancers. Another possibility is that other regulatory elements, located outside the promoter region tested in this study, could influence *ERBB2* expression in colon and ovary cancer cells. For instance, sequences described in the first intron (Newman *et al*, 2000) or 12 kb upstream the conventional start site of the *ERBB2* gene (Nezu *et al*, 1999) should be studied. We also have to keep in mind the multiple levels of *erb*B-2 mRNA and protein expression regulation. Indeed, multiple *erb*B-2 transcripts have been described which give rise to different proteins (Scott *et al*, 1993; Doherty *et al*, 1999a, 1999b; Siegel *et al*, 1999). Moreover, p185^c-*erb*B-2^ is proteolytically cleaved to generate a soluble extracellular fragment and a constitutively active intracellular fragment (Zabrecky *et al*, 1991; Pupa *et al*, 1993; Esparis-Ogando *et al*, 1999). No single detection method is able to pick up all these important variants.

In summary, this study is, to our knowledge, the first attempt to understand the mechanisms of *ERBB2* overexpression in non-breast cancer cells. In these cells, the gene copy number is normal and the overexpression is moderate. Our results suggest that different mechanisms lead to *ERBB2* overexpression in breast and in non-breast cancers. In contrast to breast cancer cells, in colon cancers the protein and transcript levels are not correlated, suggesting a regulation at the levels of translation and/or post-translation. One of the main finding is that AP-2 transcription factors are probably not involved in the increase in *erb*B-2 transcript levels in tumours of non-breast origins. Fragments of the *ERBB2* promote act differently in breast and in ovary or colon cancer cells we have analysed. Additional studies are needed to understand the mechanisms responsible for the increased accumulation of the *erb*B-2 transcript and protein in non-breast cancer cells.
